# Natural history of non-polyglutamine *CACNA1A* disease in Austria

**DOI:** 10.1007/s00415-024-12602-y

**Published:** 2024-08-07

**Authors:** Elisabetta Indelicato, Wolfgang Nachbauer, Matthias S. Amprosi, Sarah Maier, Iris Unterberger, Margarete Delazer, Katharina Kaltseis, Stefan Kiechl, Gregor Broessner, Matthias Baumann, Sylvia Boesch

**Affiliations:** 1 Center for Rare Movement Disorders Innsbruck, Department of Neurology, Medical University of Innsbruck, Anichstrasse 35, 6020 Innsbruck, Austria; 2 Public Health, Health Economics, Medical Statistics and Informatics, Institute of Clinical Epidemiology, Innsbruck, Austria; 3 Epilepsy Center, Department of Neurology, Medical University of Innsbruck, Innsbruck, Austria; 4 Neuropsychology, Department of Neurology, Medical University of Innsbruck, Innsbruck, Austria; 5 Headache Outpatient Clinic, Department of Neurology, Medical University of Innsbruck, Innsbruck, Austria; 6 VASCage, Centre on Clinical Stroke Research, Innsbruck, Austria; 7 Department of Pediatrics I, Medical University of Innsbruck, Innsbruck, Austria

**Keywords:** *CACNA1A*, Hemiplegic migraine, Episodic ataxia, Natural history, Outcome measurements, Anti-CGRP antibody

## Abstract

**Background and objectives:**

Non-polyglutamine *CACNA1A* variants underlie an extremely variable phenotypic spectrum encompassing developmental delay, hemiplegic migraine, epilepsy, psychiatric symptoms, episodic and chronic cerebellar signs. We provide our experience with the long-term follow-up of *CACNA1A* patients and their response to interval therapy.

**Methods:**

Patients with genetically confirmed non-polyglutamine *CACNA1A* disease were prospectively followed at the Center for Rare Movement Disorders of the Medical University of Innsbruck from 2004 to 2024.

**Results:**

We recruited 41 subjects with non-polyglutamine *CACNA1A* disease, of which 38 (93%) familial cases. The mean age at the first examination was 35 ± 22 years. Disease onset was in the childhood/adolescence in 31/41 patients (76%). Developmental delay and episodic symptoms were the first disease manifestation in 9/41 (22%) and 32/41 (78%) patients respectively. Chronic neurological signs encompassed a cerebellar syndrome in 35/41 (85%), which showed almost no progression during the observation period, as well as cognitive deficits in 9/20 (45%, MOCA test score < 26), psychiatric and behavioral symptoms in 11/41(27%). Seizures occurred in two patients concomitant to severe hemiplegic migraine. At the last visit, 27/41 patients (66%) required an interval prophylaxis (including acetazolamide, flunarizine, 4-aminopyridine, topiramate), which was efficacious in reducing the frequency and severity of episodic symptoms in all cases. In one patient in his 70ies with progressively therapy resistant hemiplegic migraine, treatment with the anti-CGRP antibody galcanezumab successfully reduced the frequency of migraine days from 4 to 1/month.

**Conclusions:**

Non-polyglutamine *CACNA1A* disease show an evolving age-dependent presentation. Interval prophylaxis is effective in reducing the burden of episodic symptoms.

## Introduction

*CACNA1A* encodes the pore-forming subunit of the voltage-gated calcium channel P/Q [1].

Heterozygous variants in *CACNA1A* were historically associated with three well-defined neurological phenotypes: familial hemiplegic migraine type 1 (FHM1), episodic ataxia type 2 (EA2), and spinocerebellar ataxia type 6 (SCA6) [2, 3]. FHM1 is a monogenic form of migraine mainly characterized by prominent motor deficits during the aura and it is associated with missense *CACNA1A* variants [4]. EA2 presents with recurring spells of vertigo, balance worsening and oscillopsia and is most commonly caused by truncating/frameshift variants [5]. SCA6 is caused by a pathologically expanded polyglutamine-stretch. It may feature episodic symptoms at disease onset, but its main manifestation is a late onset, progressive, isolated cerebellar syndrome [6].

The clinical presentation of non-polyglutamine *CACNA1A* disorders may be extremely variable [7] and show a substantial overlap between FHM1 and EA2 [8]. Cumulative research highlighted that chronic cerebellar signs develop during the disease course in a majority of cases [8]. Detailed analysis of multigenerational pedigrees also revealed a high frequency of early onset symptoms in the offspring such as developmental delay, epilepsy, learning difficulties and behavioral issues [8, 9]. The advent of unbiased whole exome sequencing further expanded the clinical spectrum associated with *CACNA1A* variants. Large screening studies in early-onset severe neurodevelopmental phenotypes including congenital ataxia and epileptic encephalopathy revealed the contribution of de novo non-polyglutamine *CACNA1A* variants [10, 11]. Long-term natural history data are lacking, but sparse reports suggest that at least some of these severe early-onset cases may show a positive evolution when growing up [9, 12]. Lumping up these findings, the picture of an age-dependent phenotypic continuum emerges [8].

*CACNA1A* diseases are rare neurogenetic conditions, but they are increasingly recognized among the most common inherited cerebellar disorders [7]. Because of their multifaceted clinical presentation, they may be seen by different specialists: from movement disorders to epilepsy and headache specialists. This variability is reflected in the literature in which the characteristics of reported cohorts reflect the referral pattern of the study site [4, 5, 13, 14]. In addition to the extreme phenotypic variability, the large number of variants of uncertain significance (VUS) makes the diagnosis of *CACNA1A* disease challenging [15].

Herein, we reported the clinical characteristics of a *CACNA1A* cohort recruited through multiple channels (adult movement disorder clinic, epilepsy and headache outpatient clinic, pediatric clinic) at a single institution. We share our experience with treatment strategies in the long-term follow-up, including a first report of efficacy and tolerability of an anti-CGRP monoclonal antibody in FHM1.

## Methods

### Subject recruitment and evaluation

Genetically confirmed *CACNA1A* patients were prospectively followed either at the Department of Neurology or at the Department of Pediatrics of the Medical University of Innsbruck from 2004 until 2024. Genetic testing consisted of targeted *CACNA1A* sequencing or exome-based panel diagnostics. Variants were classified according to the criteria of the American College of Medical Genetics and Genomics (ACMG) [16]. We re-assessed population frequencies based on the latest release of gnomAD (version 4.1) and used a machine learning based method (https://funnc.shinyapps.io/shinyappweb/) for computational prediction of variant effect [17]. At the time of the first referral and during the follow-up we collected clinical information concerning: age at onset, clinical findings, frequency and expression of the episodic manifestations, ongoing medical therapy, and its impact on the episodic symptoms. We defined the age at onset as the age at which whichever symptom suggestive of *CACNA1A* disease (developmental delay or episodic manifestations or chronic ataxia) became clinically manifest. The present study was approved by the local institutional review board (Study Number 1022/2020). Thirty-one patients had been included in previous reports [7, 9, 12], while 10 subjects are described here for the first time. Hemiplegic migraine was diagnosed according to the ICHD-3 criteria.

### Outcome measurements

The Scale for Assessment and Rating of Ataxia (SARA) was applied to monitor the severity of the chronic cerebellar syndrome in adults [18]. Instrumented gait analysis was performed by means of wearable sensors (Portabiles HealthCare Technologies GmbH, Erlangen). We applied the Montreal Cognitive Assessment (MoCA) test as a screening tool for cognitive impairment [19]. To assess impairment in daily functioning, we applied the Unified Huntington´s Disease Rating Scale Part IV (UHDRS-IV) and the Activity of Daily Living (ADL) inventory of the modified Friedreich´s Ataxia Rating Scale based on other natural history studies in cerebellar disorders [20–22]. We included also the UHDRS-IV as in a natural history studies in spinocerebellar ataxias [20, 21], since it also cover limitations in daily functioning due to psychiatric/behavioral issues. Anxiety was assessed by means of the General Anxiety Disorder-7 (GAD-7) questionnaire [23]. To quantify the burden associated to the episodic symptoms, we developed a paroxysmal symptoms inventory, addressing eight specific domains (speech disturbances, motor, sensory, balance, visual symptoms, vegetative symptoms, consciousness, psychiatric issues, headache). Patients were asked (i) to assign a severity score based on the visual analogue scale (VAS) to each symptom and subsequently (ii) to describe in detail the symptom features.

### Statistics

We reported descriptive statistics of the collected clinical features using mean ± standard deviation, median with interquartile ranges and percentages. To compare subgroups we applied the Fisher exact test and the Mann–Whitney U test or the t-test depending on the variable distribution. Normality was tested by means of Kolmogorow-Smirnow test or Shapiro–Wilk test. The progression of SARA during the follow-up was analysed using a linear mixed model and illustrated using the estimated marginal means with their standard error.

## Results

### *CACNA1A* cohort characteristics

**Fig. 1:**
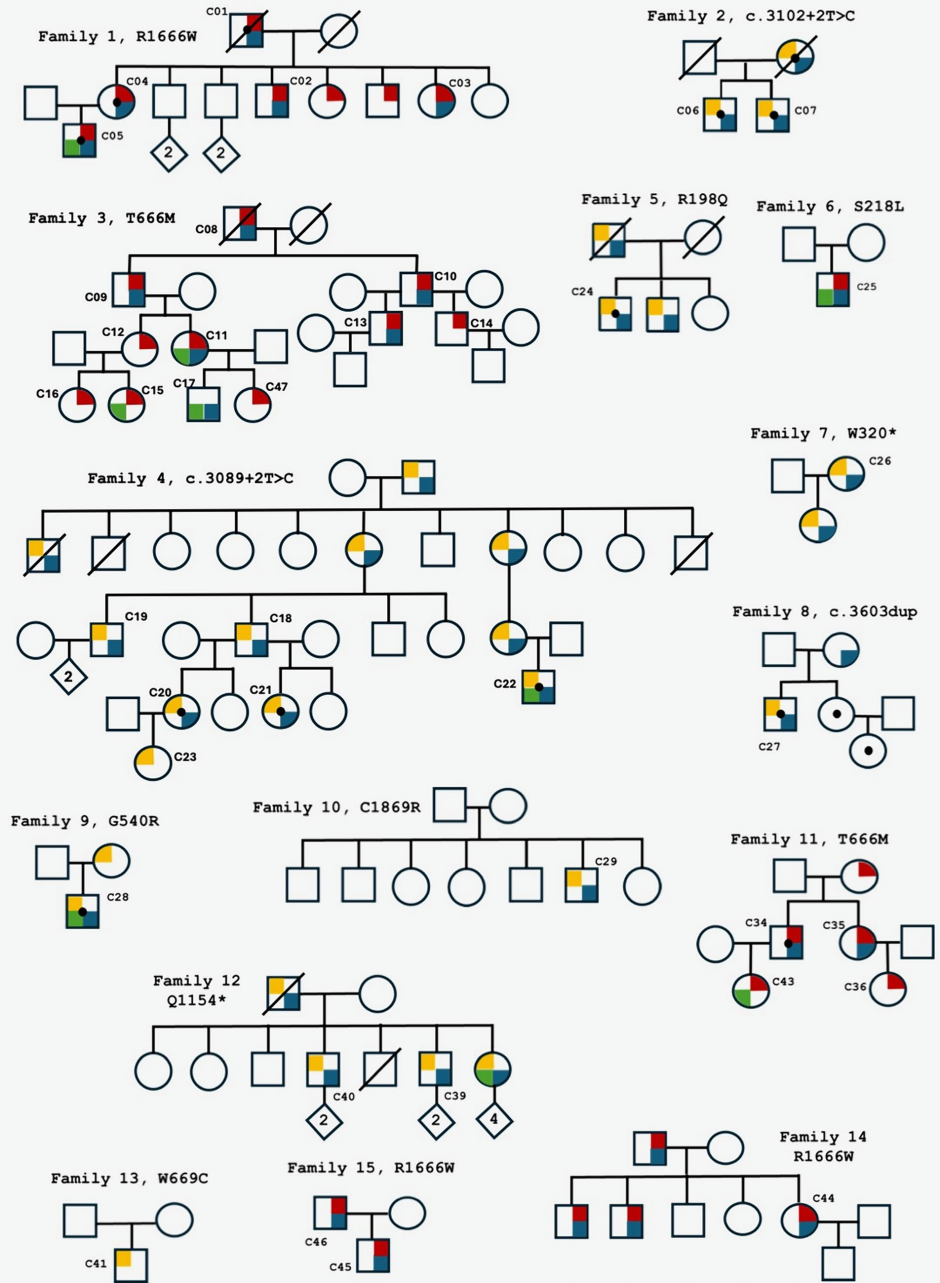
Family trees of the Innsbruck *CACNA1A* cohort. Blue quadrant: chronic gait ataxia, green quadrant: developmental delay, red quadrant: hemiplegic migraine, yellow quadrant: episodic ataxia. Black dot: psychiatric features

Between 2004 and 2024, 41 subjects with non-polyglutamine *CACNA1A* variants (16 females, 39%, see also Fig. 1 and Table 1) were followed in Innsbruck. Thirty-eight cases occurred within families with more than one affected subject. Three patients were simplex cases; trio analysis was performed in two of them revealing a de novo origin of the carried S218L and W669C variant. Twelve different variants were detected of which 2 recurring ones (T666M, R1666W). All variants were classified as likely pathogenic or pathogenic according to the ACMG criteria at the time of diagnosis. At that time, none of the variants were present in population databases (moderate supportive criterion “PM2”). Interestingly, the variant R1666W, which has been repeatedly reported in association with FHM1 [4, 7], is now found in two out of 1,613,898 alleles in the latest release of gnomAD (v4.1, April 2024). In the absence of the PM2 criterion, the R1666W variant should now be reclassified as a variant of uncertain significance (VUS). However, variable expressivity has been reported for this variant, with carriers presenting only with migraine without aura [4]. Given the reduced expressivity, it may be possible to find two carriers in > 1 million control chromosomes. It is noteworthy that the number of alleles in gnomAD v4 has increased almost fivefold compared to the previous version v2, an issue that also challenges variant interpretation in other neurogenetic disorders [24].

**Table 1:** Clinicogenetic characteristics of the Innsbruck *CACNA1A* cohort

Family	Variant	ClinVar	Current ACMG variant classification	ID	Sex	Onset	First manifestation	Interval therapy
1	**R1666W** Transcript NM_001127222.2	Pathogenic/likely pathogenic	VUS* (PM1, PP1, PP3,PP5)	C01	Male	Adulthood	HM	
				C02	Male	School age	HM	Topiramate
				C03	Female	Adulthood	HM	4-aminopyridine
				C04	Female	Adulthood	HM	Acetazolamide
				C05	Male	Infancy	DD	Acetazolamide
2	**c.3102 + 2 T > C** Transcript NM_001127221.1		Pathogenic (PVS1, PM2, PP1)	C06	Male	Infancy	EA	Acetazolamide
				C07	Male	Adulthood	EA	Acetazolamide
3	**T666M** Transcript NM_001127221.2	Pathogenic	Pathogenic (PS3, PM1-2, PP1, PP3, PP5)	C08	Male	Adulthood	HM	Flunarizine
				C09	Male	Preschool age	HM	Galcanezumab
				C10	Male	Adolescence	HM	Flunarizine
				C11	Female	Infancy	DD	
				C12	Female	School age	HM	
				C13	Male	Preschool age	HM	Topiramate
				C14	Male	School age	HM	
				C15	Female	Toddler	DD	
				**C16**	Female	School age	HM	
				C17	Male	Toddler	DD	
				**C47**	Female	School age	HM	
4	**c.3089 + 2 T > C** Transcript NM_001127221.1		Pathogenic (PVS1, PM2, PP1)	C18	Male	School age	EA	4-aminopyridine
				C19	Male	Adulthood	EA	Acetazolamide
				C20	Female	Toddler	EA	Acetazolamide
				C21	female	School age	EA	4-aminopyridine
				C22	Male	Infancy	DD	Acetazolamide
				**C23**	Female	Infancy	EA	
5	**R198Q** Transcript NM_001127221.2	Likely pathogenic/uncertain significance	Likely pathogenic (PM1-2, PP3, PP5)	C24	Male	Infancy	EA	Acetazolamide
6	**S218L** Transcript NM_001127221.2	Pathogenic/likely pathogenic	Pathogenic (PS3, PM1-2, PM6, PP3; PP5)	C25	Male	Infancy	DD	Flunarizine + acetazolamide
7	W320* Transcript NM_001127221.1		Pathogenic (PVS1, PM1-2, PP1, PP3)	C26	Female	School age	EA	Acetazolamide
8	**c.3603dup** Transcript NM_001127221.1		Pathogenic (PVS1, PM1-2)	C27	Male	School age	EA	Acetazolamide
9	**G540R** Transcript NM_001127221.2	Pathogenic/ uncertain significance	Likely pathogenic (PS3, PM1-2, PP1)	C28	Male	Toddler	DD	Acetazolamide
10	**C1869R** Transcript NM_001127221.2	Not provided	Likely pathogenic (PM1-2, PP3, PP5)	C29	Male	Adulthood	EA	4-aminopyridine
11	**T666M** Transcript NM_001127221.2	Pathogenic	Pathogenic (PS3, PM1-2, PP1, PP3, PP5)	**C34**	Male	Toddler	HM	Acetazolamide
				**C35**	Female	School age	HM	Flunarizine
				**C36**	Female	School age	HM	
				**C43**	Female	Infancy	DD	
12	Q1154* Transcript NM_001127221.1	Pathogenic	Pathogenic (PVS1, PM2, PP1)	C38	Female	Toddler	DD	Acetazolamide
				C39	Male	Adulthood	EA	4-aminopyridine
				C40	Male	Adulthood	EA	Acetazolamide
13	**W669C** Transcript NM_001127222.2		Likely pathogenic (PM1-2, PM6, PP3-4)	C41	Male	School age	EA	4-aminopyridine
14	**R1666W** Transcript NM_001127222.2	Pathogenic/likely pathogenic	VUS* (PM1, PP1, PP3,PP5)	**C44**	Female	Adulthood	HM	
15	**R1666W** Transcript NM_001127222.2	Pathogenic/likely pathogenic	VUS* (PM1, PP1, PP3,PP5)	**C45**	Male	School age	HM	
				**C46**	Male	School age	HM	

The IDs of patients which are reported here for the first time are marked in bold. The third column from the left shows the variants annotated in ClinVar (last access June 30, 2024) and their classification. The fourth column shows the current variant classification according to the ACMG criteria: for the interpretation of the variant R1666W see also the details in the main text

*EA* episodic ataxia; *HM* hemiplegic migraine; *DD* developmental delay; *VUS* variant of uncertain significance

**Fig. 2:**
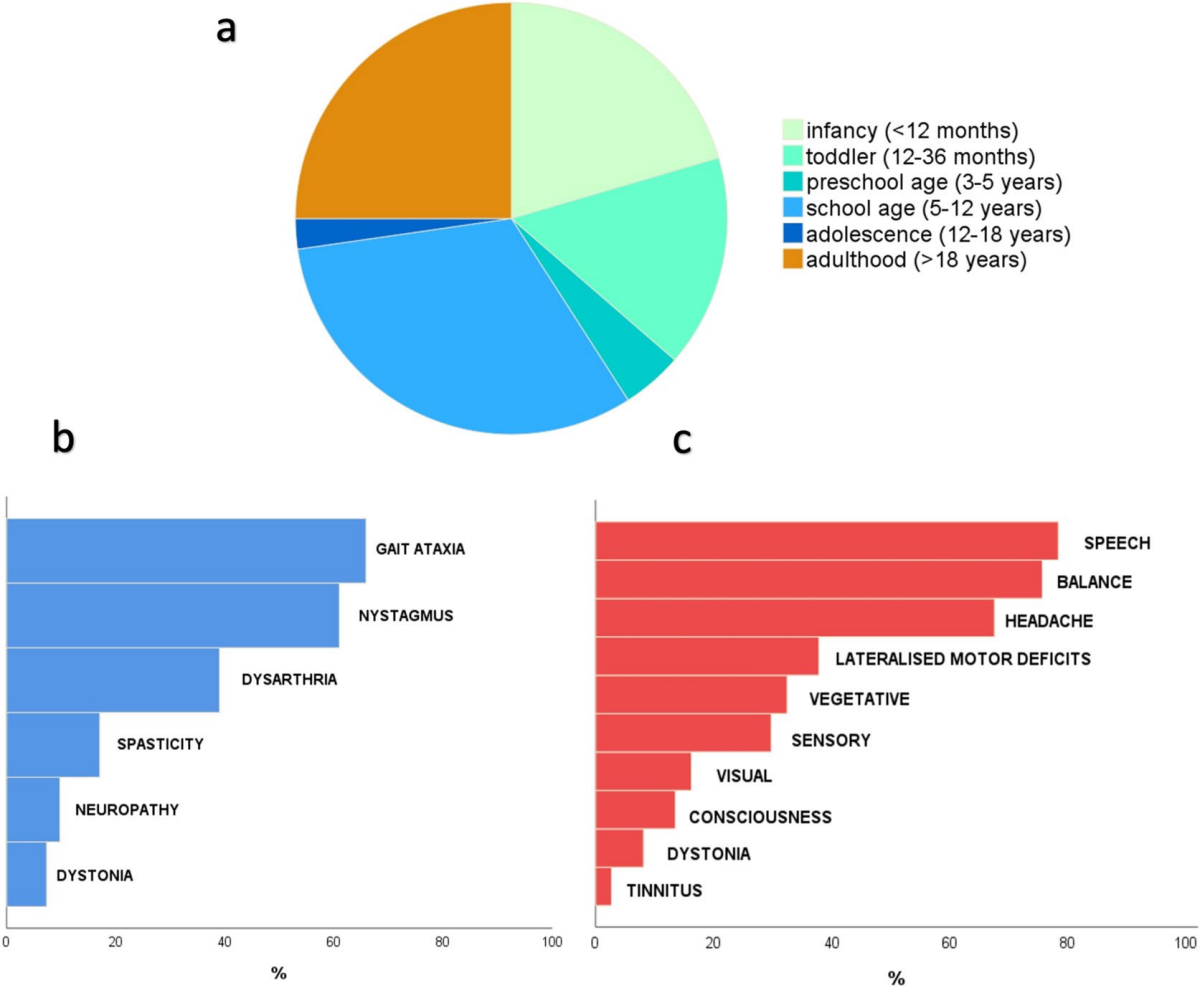
Clinical characteristics of the Innsbruck *CACNA1A* cohort. **a** Distribution of Age at Onset; **b** Frequency of chronic neurological signs; **c** Frequency of episodic symptoms

The mean age at the first examination at our center was 35 ± 22 years. Twenty-four patients (59%) were classified as having FHM1, the other 17 patients (41%) as having EA2. Disease onset was in the childhood/adolescence in 31 patients (76%; see for details Fig. 2A). A developmental delay was the first sign in nine patients, while in the other 32 the first manifestation were episodic symptoms. Eventually, almost all patients (n = 40, 98%) reported episodic symptoms during the disease course. Episodic symptoms were the only manifestation of the disease in merely 6 young patients (15%, mean age 9 ± 7 years).

### Chronic neurological signs

Thirty-five patients (85%) displayed chronic cerebellar signs, specifically gait ataxia (28/41, 68%), followed by nystagmus (24/41, 56%) and dysarthria (15/41, 37%). In most patients the cerebellar syndrome was mild and remained stable during the clinical follow-up. In 10/41 patients (24%) chronic ataxia was progressive and dominated the clinical picture. Cerebellar atrophy, mostly mild and more pronounced in the vermis, was detected in 30 out of 36 patients (83%) who underwent brain MRI at our center. Further encountered chronic manifestations were spasticity (6/41, 15%), peripheral neuropathy (3/41, 7%) and dystonia (3/41, 7%). Eleven patients (27%) suffered from psychiatric comorbidities (anxiety, depression, adaptation disorder, attention deficit hyperactivity disorder, personality disorder, schizophrenia).

Patients with chronic gait ataxia were older (age at examination 43 ± 20 years versus 16 ± 13 in those without gait ataxia, *p* < 0.001). Presence of peripheral neuropathy was specific to older patients (age at examination 67 ± 4 years versus 32 ± 21 in those without neuropathy, *p* = 0.009). Dystonia as chronic sign was also more frequent in older patients (59 ± 13 versus 33 ± 21, *p* = 0.054). Nystagmus did not show an age-dependency.

### Paroxysmal manifestations

The most common neurological symptoms during attacks were speech disturbances (28/40, 70%) followed by emerging/worsening gait instability (25/40 patients, 63%) and headache (25/40, 63%). Further paroxysmal symptoms listed in descending frequency were nausea (13/40, 33%), hemiparesis (33%), paresthesia (9/40, 23%), visual disturbances (8/40, 20%), impaired consciousness (5/40, 13%), dystonia (4/40, 10%) and tinnitus (1/40).

The frequency of paroxysmal manifestations was variable with ≥ 1 episode/week in 10/40 patients, with ≥ 1 episode/month in other 10/40 patients, < 1/month—≥ 1 episode/year in 15/40, while 5/40 reported < 1 episode/year or even one single episode. Paroxysmal manifestations last from minutes in patients with EA2 phenotype up to hours and days (mostly in FHM1 patients).

Nineteen patients reported triggers for the episodic manifestations. The most frequent were stress (7 EA2, 4 FHM1) caffeine (7 EA2), anxiety/agitation (4 EA2), physical exertion (4 EA2), alcohol (3 EA2), fever/heat (1 EA2), weather changes (2 FHM1), trivial head trauma (1 FHM1, 1 EA2). In two FHM1 patients (C04, C08) an episode of hemiplegic migraine was triggered by cerebral angiography.

A seizure had been observed in one patient (C25) during a severe hemiplegic migraine attack with brain edema harboring the S218L variant. The remaining patients had no convincing history of epilepsy at the time of referral and during the follow-up.

In a child harboring the variant T666M (C47), the first migraine episode presented as a meningoencephalitis (fever, vomiting, meningismus, disturbance of consciousness) with brain MRI showing a left dural enhancement and left hemispheric slowing in EEG.

### Differences and overlap in EA2 and FHM1

In the comparative analysis, age at onset did not differ between individuals with EA2 and FHM1 (*p* = 0.990). Both groups showed a similar frequency of chronic cerebellar signs and positive history for psychomotor delay (both *p* = 1.000). Paroxysmal manifestations were markedly more frequent in patients with EA2 (Median 108/year (IQR 24–216) versus 6.5/year (IQR 1–12) in FHM1, *p* < 0.001). The duration of paroxysmal manifestations was generally shorter in EA2 compared to FHM1, but highly variable. Paroxysmal vertigo (*p* < 0.001) and gait ataxia (*p* = 0.002) were more frequent in EA2. Lateralized motor deficits and headache occurred more frequently in FHM1 patients (both *p* < 0.001). Impaired consciousness occurred only in patients with FHM1 (n = 5; *p* = 0.065). FHM1 and EA2 showed a similar frequency of speech disturbances, visual disturbances, paresthesia and paroxysmal dystonia during their spells. Concerning the chronic manifestations, both cerebellar and non-cerebellar signs had a similar frequency in FHM1 and EA2 patients. Dystonia as a chronic sign was encountered exclusively in EA2 patients (n = 3, *p* = 0.064). Psychiatric comorbidities were more frequent in the EA2 group (8/17 patients versus 3/24 patients in FHM1, *p* = 0.029).

### Outcome measurements

**Fig. 3:**
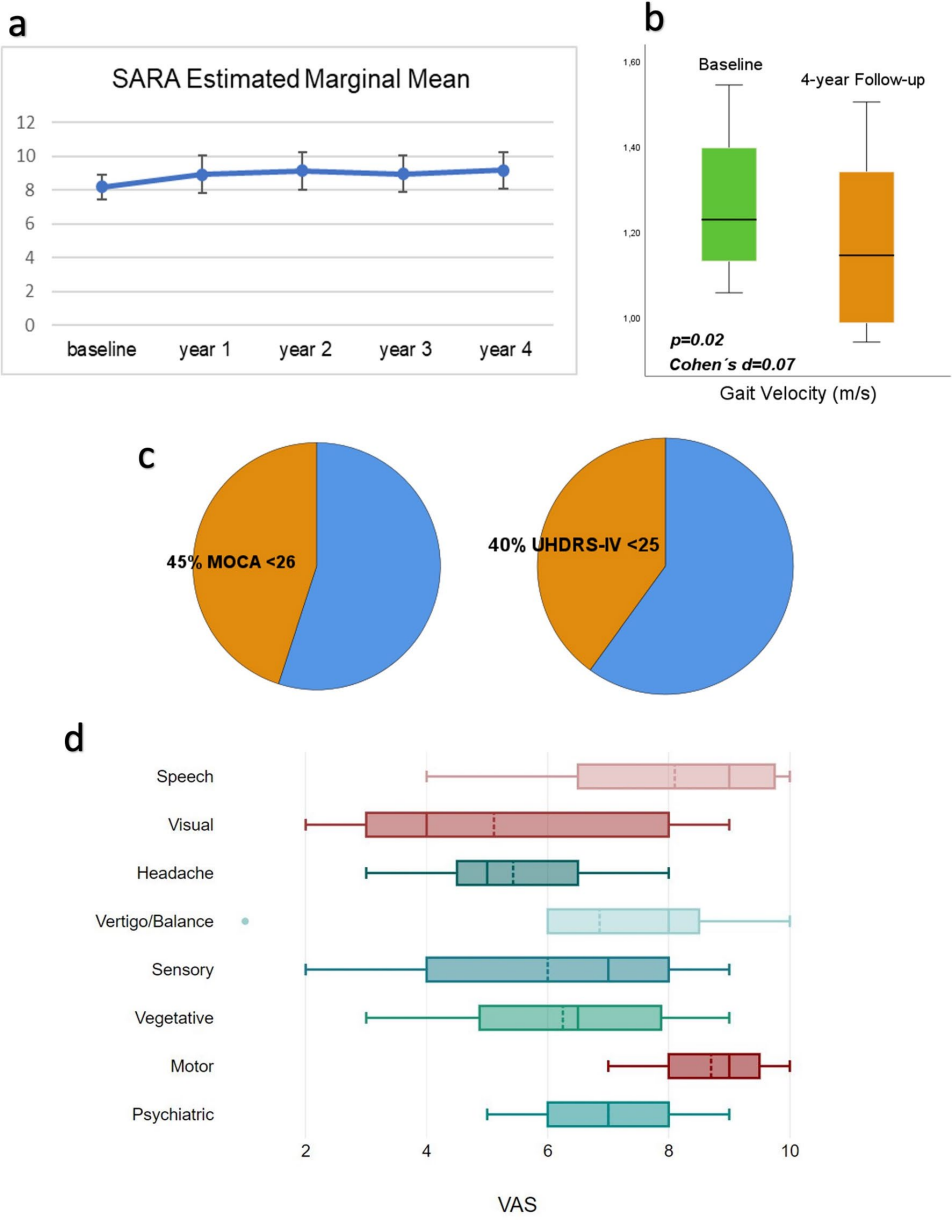
Outcome measurements in the Innsbruck *CACNA1A* cohort. **a** Evolution of SARA scores over 4 years, **b** Gait velocity as measured by instrumented gait analysis in a 4-year interval; **c** Frequency of pathological findings at MOCA and UHDRS-IV; **d** Burden of the episodic symptoms as captured by the dedicated questionnaire

We collected the SARA score in 29 patients at the first visit. Mean SARA score was of 8 ± 4, corresponding to an overt ataxia, but with still independent gait. We analysed the evolution of SARA in 21 patients for whom regular follow-ups were completed over a 4-year interval. There was no significant progression in SARA (*p* = 0.919, see Fig. 3a). Adjusting for age at onset and age at SARA score assessment did not improve the model. In 12 patients undergoing instrumented gait analysis in an interval of four years, a significant change was found only concerning gait velocity (1.115 ± 0.219 m/s at follow-up versus 1.228 ± 0.152 m/s at baseline, *p* = 0.020, see Fig. 3b). A MOCA score < 26 was found in 9/20 Patients (Median MOCA score 27, IQR 21–27, see Fig. 3c). The most affected subitem of MOCA was verbal fluency. Median UDHRS-IV score was 25 (IQR 23–25) with 8/20 patients with impairment in at least one domain of the scale (see Fig. 3c). Median FARS-ADL was 2 (IQR 1–6). Mean GAD-7 score was 5 ± 3. At the time of the last visit, 12 patients reported episodic symptoms in the previous four weeks. None of them required hospitalization. In the VAS-based questionnaire, the most bothersome issue were speech disturbances and motor deficits. Speech issues receiving 9–10 VAS were described as anarthria with additional swallowing difficulties or complete motor aphasia. Hemiparesis/Hemiplegia was also assigned 9–10 VAS by patients. A detailed summary is offered in Fig. 3d.

### Course of the episodic manifestations and medical therapy

At the time of the last visit, 27 patients required a preventative medication, which comprised acetazolamide (n = 14), 4-aminopyridine (n = 6), flunarizine (n = 3), topiramate (n = 2), a combination of flunarizine and acetazolamide (n = 1) and galcanezumab (n = 1). Generally, preventative medication was effective in drastically reducing the frequency and severity of the episodic symptoms. Despite some patients reporting freedom from spells, cross-check with the clinical chart (including non-reported emergency room referrals) and interview with family members suggested that an episode-free course was the exception. Indeed, most patients went on experiencing what they defined “smaller spells”, the frequency of which (through cross-check with clinical charts) was probably underestimated. In five adult FHM1 patients clinical history and chart review indicated a two-peak course of paroxysmal manifestations with occurrence of hemiplegic migraine in the youth, a consecutive migraine-free interval of several years and reoccurrence of hemiplegic migraine beyond their forties. Two of them experienced a particularly severe relapse in their sixties with 4–5 migraine days/month with hemiplegic aura. One patient tried acetazolamide and flunarizine with no benefit. He finally experienced a reduction of migraine days from 4/month to 1/bimonthly under topiramate 100 mg/daily. The other patient underwent trials with acetazolamide (not tolerated), flunarizine, bisoprolol and candesartan. Considering the clinical situation (67 years old, mild cognitive impairment) topiramate was not tried in this case. After consultation of a headache specialist and confirmation of the episodic migraine diagnosis, the patient was started on the Anti-CGRP monoclonal antibody galcanezumab (starting dose 2 × 120 mg followed by 120 mg s.c. monthly), which was well tolerated. At 6-month follow-up, he reported a reduction of migraine days from 4/month to 1/month under galcanezumab from the first month onwards.

## Discussion

We report the clinical features of 41 patients with non-polyglutamine *CACNA1A* disease from a national reference center for rare neurological diseases in Austria.

The age at onset in our cohort was extremely variable, as reported in a recent multicentric study [7]. The majority of patients however showed the first signs of *CACNA1A* disease, consisting of developmental delay or episodic symptoms, early in life. Almost all patients developed in the course of the disease chronic cerebellar signs. Further non-episodic features encompassed psychiatric symptoms, spasticity, and dystonia. All patient but one child displayed episodic symptoms in the form of hemiplegic migraine or episodic ataxia, with symptoms often overlapping between the two. The frequency of episodic symptoms was extremely variable, but high enough to require an interval therapy in 66% of patients. The most frequently applied therapies were acetazolamide, irrespectively of the phenotype, and 4-aminopyridine in patients with EA2. The interval therapy was effective in all patients in reducing the frequency and severity of the episodic symptoms, but freedom from episodic features was the exception. As previously noticed [9, 12], several patients could not recall episodes at the time of their visit, but their occurrence was documented in hospital charts and emergency room accesses. The treatment of a hemiplegic migraine relapse in older FHM1 patients posed challenges. In one case with relapse of hemiplegic migraine in the 7th decade, the exploitation of conventional antimigraine drugs motivated a trial with the anti-CGRP monoclonal antibody galcanezumab. Patients with hemiplegic migraine in general have been excluded by protocol from all pivotal trials with anti-CGRP antibodies. Anecdotal reports in sporadic hemiplegic migraine suggest that they are safe and potentially effective [25]. To the best of our knowledge, this is the first report of a successful application of such a therapeutic strategy in patients with genetically confirmed *CACNA1A* disease. Although we report on a single patient, this case sets a precedent which may support a trial with this effective therapy in the setting of therapy resistant *CACNA1A*-associated hemiplegic migraine.

Several patients series with *CACNA1A* variants have been published [4, 5, 7, 13–15, 26], including three large cohorts in the last few years [7, 13, 14]. Comparing to a recent, very large, multicentric study with n = 239 cases [7], our single-center view allowed a long follow-up with analysis of the disease evolution and therapy response in a real-life setting and might be thus more informative for clinicians. Two other multicentric cohorts with n = 47 patients each, reported a much higher percentage of severe, early onset phenotype and epilepsy. One cohort recruited in several Israeli and European pediatric centers focused only on infantile onset cases [13]. The other multicentric cohort from North America was recruited through a patient support organization with parents or patients filling up an online questionnaire and no cross-check with clinical charts and may be thus skewed to more severely impacted individuals [14]. This might particularly be the case in the setting of de novo variants which indeed represented more than the half of the detected variants in the North American study [14] versus our cohort with mostly familial cases. Since our cohort comprised mostly adults, we might have undergone the opposite bias. Earlier *CACNA1A* cohorts included specifically patients with episodic ataxia or hemiplegic migraine phenotype [4, 5, 26]. In the present study, patients were recruited at a university hospital covering multiple specialties including child neurology, epileptology, headache clinic, and thus are more likely to reflect the real distribution of clinical phenotypes in a *CACNA1A* cohort at a given time point in our region. Children with severe phenotypes may still cover development milestones and show rather mild deficits in the adulthood [9, 12]. In adult patients, recall of early symptoms may be subjected to bias. These issues may lead to underestimate the frequency of early features such as developmental delay.

To inform the design of future trials, we collected several outcome measurements. Our clinical experience shows that chronic ataxia may be progressive and become the greatest burden in older patients. Nonetheless, the available rating tool (SARA, instrumented gait analysis), were not able to capture a substantial evolution of chronic ataxia in our cohort. Beyond the heterogeneity in disease trajectories and the overall slow progression, also the occurrence of prominent fluctuations likely contributes to the poor performance of available clinician reported outcome measurements. The impairment due to cognitive and behavioral symptoms, as captured by MOCA, UHDR-IV and GAD-7, highlights the need for composite outcome measurements covering both motor and non-motor features. In a questionnaire on episodic symptoms patients pinpointed speech and motor disturbances as the greatest determinants of burden. Our detailed information may guide the clinical examination towards the suspicion of *CACNA1A* disease and inform the compilation of a *CACNA1A* specific patient reported outcome measurement.

In conclusion, our report describes the natural history and therapy response of a large monocentric *CACNA1A* cohort, providing valuable information for the clinicians involved in the monitoring, counseling and treatment of these disorders. We provide single-patient data supporting the evaluation of anti-CGRP antibodies as alternative strategy in CACNA1A-related therapy refractory hemiplegic migraine.

## Data Availability

Patient informed consent in the present study did not include the transfer of data in a publicly available repository. Raw data are available from the first author or corresponding author upon request.
